# Stereotyped Spatiotemporal Dynamics of Spontaneous Activity in Visual Cortex Prior to Eye Opening

**DOI:** 10.1523/JNEUROSCI.1420-24.2025

**Published:** 2025-04-22

**Authors:** Luna Kettlewell, Audrey Sederberg, Gordon B. Smith

**Affiliations:** ^1^Department of Neuroscience, University of Minnesota, Minneapolis, Minnesota 55455; ^2^Graduate Program in Neuroscience, University of Minnesota, Minneapolis, Minnesota 55455; ^3^Optical Imaging and Brain Sciences Medical Discovery Team, University of Minnesota, Minneapolis, Minnesota 55455

**Keywords:** calcium imaging, development, ferret, networks, spatiotemporal, visual cortex

## Abstract

Over the course of development, functional sensory representations emerge in the visual cortex. Prior to eye opening, modular patterns of spontaneous activity form long-range networks that may serve as a precursor for mature network organization. Although the spatial structure of these networks has been well studied, their temporal features, which may contribute to their continued plasticity and development, remain largely uncharacterized. To address this, we imaged hours of spontaneous network activity in the visual cortex of developing ferrets of both sexes utilizing a fast calcium indicator (GCaMP8m) and wide-field imaging at high temporal resolution (50 Hz). The spatial structure of this activity was highly modular, exhibiting distributed and spatially segregated active domains and long-range correlated networks on the timescale of tens of milliseconds. We found that the majority of events showed a clear dynamic component in which the patterns of active modules shifted over the course of events lasting a few hundred milliseconds. Although only a minority of events were well fit with a linear traveling wave, more complex spatiotemporal patterns occurred in repeated and stereotyped motifs across hours of imaging. Finally, we found that the most frequently occurring single-frame spatial activity patterns were predictive of future activity and separable spatiotemporal trajectories extending over many hundreds of milliseconds. Together, our results demonstrate that spontaneous activity in the early developing cortex exhibits a stereotyped spatiotemporal structure on fast timescales, suggesting a potential role in the maturation and refinement of future functional representations.

## Significance Statement

Understanding the temporal dynamics underlying the network structure in early development is critical for understanding network function and plasticity. By imaging hours of spontaneous cortical activity, we find evidence that the majority of spontaneous neural activity is dynamic with repeated and complex spatiotemporal patterns with stereotyped structure across hours. This suggests the potential for Hebbian learning in the development and refinement of functional visual representations. We also find that frequently occurring spatial activity patterns are predictive of subsequent activity for up to one second, which may indicate attractor dynamics in spontaneous activity. Our findings characterize key features of the temporal structure of spontaneous activity in the visual cortex early in development and deepen our understanding of developing neural networks.

## Introduction

During development, functional neural networks emerge in the visual cortex that exhibit selectivity for features of the visual environment. In animals such as humans, other primates, and carnivores, these networks are organized into a modular structure in which nearby neurons have similar selectivity, and groups of similarly tuned modules are distributed across the cortical surface (Hubel and Wiesel, 1997). Such modular representations have been identified for visual features such as edge orientation (Blasdel and Salama, 1986) and direction of motion (Weliky et al., 1996), among many others (Issa et al., 2000; Kara and Boyd, 2009; Smith et al., 2015b). In the ferret, selectivity for orientation and direction emerge over the course of development, with orientation preference maps first appearing around the time of eye opening [approximately postnatal day (P) 30–32; Chapman et al., 1996] and direction emerging over the subsequent week (Li et al., 2006).

Notably, the distributed and modular organization that is a hallmark of these functional maps is present in ongoing spontaneous activity at least 10 d prior to eye opening (Smith et al., 2018). These early modular patterns of millimeter-scale spontaneous activity predict features of the future orientation map but also undergo considerable refinement prior to eye opening (Smith et al., 2018). A central feature of this early spontaneous activity is the presence of long-range millimeter-scale correlations between modules. Computational and experimental results have suggested that, prior to the developmental emergence of long-range horizontal axons (Durack and Katz, 1996), such long-range correlated networks can arise through purely local interactions. Models in the form of local excitation/lateral inhibition (LE/LI) can account for both the presence of modular functional activity and millimeter-scale correlations (Smith et al., 2018) and are consistent with the ability of the early cortex to self-organize unstructured inputs (Mulholland et al., 2024b).

However, this prior work has focused on the spatial structure of activity patterns, leaving unexplored the temporal dynamics of early spontaneous activity, which likely have broad implications for developmental plasticity in the cortex. Various forms of spatiotemporal organization have been observed in the visual cortex in older animals or at different scales (Benucci et al., 2007; Huang et al., 2010; Sato et al., 2012; Siegel et al., 2012; Muller et al., 2014, 2018; Smith et al., 2015a), but the spatiotemporal structure of the millimeter-scale modular spontaneous activity seen in early development remains unknown. Here we address this by recording hours of spontaneous activity in the ferret visual cortex in animals 4–9 d before eye opening. Implementing high-speed wide-field calcium imaging and a deconvolution-based analysis, we show that nonstationary temporal dynamics underlie the vast majority of large-scale spontaneous events. Our results show that most events are poorly described by a linear traveling wave and instead exhibit more complex and nonlinear spatiotemporal dynamics. These dynamics show stereotyped sets of activity patterns that reoccur across hours of spontaneous activity. Within events, activity patterns converged to a relatively small subset of possible spatial motifs and then follow separable trajectories for many hundreds of milliseconds. Together, these results reveal for the first time the complex spatiotemporal patterns of spontaneous activity in the early developing visual cortex, demonstrating stereotyped sequences of activity that may suggest the presence of attractor-like dynamics.

## Materials and Methods

### Experimental design

#### Animals

All experimental procedures were approved by the University of Minnesota Institutional Animal Care and Use Committee and were performed in accordance with guidelines from the US National Institutes of Health. We obtained six male and female ferret kits from Marshall Farms and housed them with jills on a 16/8 h light/dark cycle. No statistical methods were used to predetermine sample sizes, but our sample sizes are similar to those reported in previous publications.

#### Viral injections

Viral injections were performed as previously described (Smith and Fitzpatrick, 2016) and were consistent with prior work (Smith et al., 2018). We expressed GCaMP8m (Zhang et al., 2021) by microinjecting AAV1 expressing hSyn.jGCaMP8m.WPRE (University of Minnesota Viral Vector and Cloning Core) into layer 2/3 of primary visual cortex (∼6–8 mm lateral, ∼1–2 mm anterior of lambda; Law et al., 1988), at P10–14, ∼10–15 d before imaging. Anesthesia was induced with isoflurane (3–4%) and maintained with isoflurane (1–2%). Buprenorphine (0.01 mg/kg) and glycopyrrolate (0.01 mg/kg) were administered intramuscularly, as well as 1:1 lidocaine/bupivacaine subcutaneously at the site of incision. Animal temperature was maintained at ∼37°C with a water pump heat therapy pad (Adroit Medical HTP-1500, Parkland Scientific). Animals were mechanically ventilated and both heart rate and end-tidal CO2 were monitored throughout the surgery.

Using aseptic surgical technique, skin and muscle overlying visual cortex were retracted, and a small burr hole was made with a handheld drill (Foredom Electric). Approximately 1 µl of virus contained in a pulled-glass pipette was pressure injected into the cortex at two depths (∼200 and 400 µm below the surface) over 20 min using a Nanoject-II (World Precision Instruments). The craniotomy was filled with 2% agarose and sealed with a thin sterile plastic film, affixed with Vetbond. Consistent with prior work (Smith et al., 2018), this approach typically yielded relatively uniform expression over an area ∼3 mm in diameter, with gradual fall-off in expression at greater distances.

#### Cranial window surgery

On the day of experimental imaging, ferrets were anesthetized with 3–4% isoflurane. Atropine was administered subcutaneously (0.2 mg/kg). Animals were placed on a feedback-controlled heating pad to maintain an internal temperature of 37–38°C. Animals were intubated and ventilated, and isoflurane was delivered between 1 and 2% throughout the surgical procedure to maintain a surgical plane of anesthesia. An intraperitoneal catheter was placed to deliver fluids. EKG, end-tidal CO2, and internal temperature were continuously monitored during the procedure and subsequent imaging session. A 6–7 mm craniotomy was performed at the viral injection site and the dura retracted to reveal the cortex.

Five of the animals had a custom 3D printed insert that was placed directly onto the brain and craniotomy site, with one 4 mm cover glass (round, #1.5 thickness, Electron Microscopy Sciences) adhered to the bottom, and sealed to the skull using Kwik-Sil. This method was used to gently compress the underlying cortex and dampen biological motion during imaging. Alternatively, one animal used a custom titanium headplate that was affixed to the skull using Metabond and a titanium insert that was sealed with a snap-ring (5/16 inches internal retaining ring, McMaster-Carr) and a 4 mm cover glass glued to the insert. Upon completion of the surgical procedure, isoflurane was gradually reduced (0.6–0.9%) and then vecuronium bromide (0.4 mg/kg/h) mixed in an LRS 5% dextrose solution was delivered intraperitoneally to reduce motion and prevent spontaneous respiration.

#### Wide-field epifluorescence imaging

Wide-field epifluorescence imaging was performed with a sCMOS camera (Zyla 5.5, Andor) controlled by μManager (Edelstein et al., 2010). Images were acquired in global shutter configuration at 50 Hz with 4 × 4 binning to yield 640 × 540 pixels (Zyla) and additional offline 8 × 8 binning to yield 80 × 68 pixels. Spontaneous activity was captured in 10–20 min periods (sum total range, 130–165 min; *n* = 6 animals), with the animal sitting in a darkened room facing an LCD monitor displaying a black screen.

### Imaging preprocessing

#### Signal extraction for wide-field epifluorescence imaging

Motion correction was performed to correct for mild brain movement during imaging, which was done by registering each imaging frame to a reference frame. The region of interest (ROI) was manually drawn around the cortical region with strong expression and a high signal-to-noise ratio. The baseline $F_{0,\mathrm{pt}}$ for each pixel *p* and time *t* was obtained by applying a rank-order filter to the raw fluorescence trace with a 36th percentile rank and a time window of 28.5 s centered on time *t*. The rank and time window were chosen such that the baseline faithfully followed the slow trend of the fluorescence activity $\left(F_{\mathrm{pt}}\right)$. The baseline corrected spontaneous activity $r_{\mathrm{pt}}$ was calculated at each pixel *p* and time *t* as follows:

$$r_{\mathrm{pt}}=\frac{\Delta F_{\mathrm{pt}}}{F_{0,\mathrm{pt}}}=\frac{F_{\mathrm{pt}}\;\text{–}\;F_{0,\mathrm{pt}}}{F_{0,\mathrm{pt}}}.$$

#### Deconvolution

We used a deconvolution method, which we call prior frame subtraction, an approach designed to remove calcium decay and emphasize new activity. This is also referred to as numerical differentiation, as described in Stern et al. (2020). We first determined the decay rate of GCaMP8m over 20 ms, γ=0.89. Then we took the baseline corrected activity from each pixel *p* in frame *t* $\left(r_{\mathrm{pt}}\right)$ and subtracted the previous frame's activity $\left(r_{p,t-1}\right)$ multiplied by γ. The result was the deconvolved activity at each pixel and time $y_{\mathrm{pt}}$:

$$y_{\mathrm{pt}}=r_{\mathrm{pt}}-\gamma r_{p,t-1}.$$

#### Event segmentation

We first determined active pixels on each frame using a threshold for each pixel set at four standard deviations above its mean value. To remove spuriously active pixels, active pixels were required to be part of a contiguous area of at least 0.028 mm^2^, using morphological operations of binary erosion followed by binary dilation on the thresholded data. An event was then defined as any contiguously active time period with an inactive frame (defined as a frame without any active pixels) on either side. Finally, we only analyzed events that activated at least 1 mm^2^ of cortex across its entire duration, in order to focus on large spontaneous events, as in prior work (Smith et al., 2018).

### Statistical analysis

#### Spontaneous correlation patterns

To assess the spatial correlation structure of spontaneous activity on fast timescales, we first identified the maximally active frame in each event, providing a measure of the activity occurring within 20 ms. These frames were then filtered with a Gaussian spatial high-pass filter (SD: *s*_low_ = 23 µm and *s*_high_ = 194 µm). The resulting patterns were used to compute the spontaneous correlation patterns as the pairwise Pearson's correlation between all locations within the ROI and the seed point as described previously (Smith et al., 2018). Correlations of temporally summed events were computed as above after first taking the sum across all frames within the event.

#### Spatial extent of correlations

**Figure 1.**
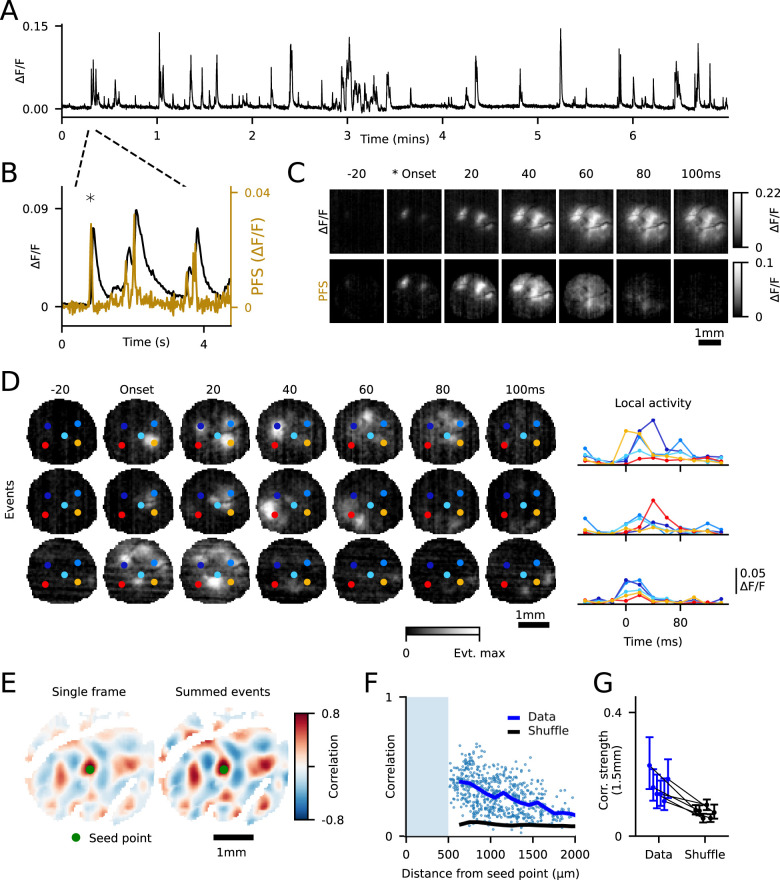
Early cortical activity exhibits fast temporal dynamics. ***A***, Spontaneous activity, averaged over field of view. ***B***, Averaged activity, zoomed in from panel ***A***. Left and right *y*-axes, respectively, show Δ*F*/*F*_0_ and a “prior frame subtraction” (PFS) deconvolution approach. ***C***, Top row shows Δ*F*/*F*_0_ and bottom row shows deconvolved activity. ***D***, Left, Three segmented events bounded on either side by inactive frames. Each event is normalized to its maximum value. Right, Averaged traces of activity within color-coded circles, highlighting dynamic activity over time. ***E***, Pixel-wise correlation patterns show distributed, modular, and long-range correlated activity at fast timescales. Left, Correlation maps computed from single frame (20 ms) per event (left) and temporally summed activity (right) show similar spatial patterns. ROIs were drawn to exclude large blood vessels. ***F***, Correlation strength as a function of distance from the seed point for single-frame correlations shown in ***E***, compared against shuffled control (see Materials and Methods). ***G***, Long-range single-frame correlations (1.5 mm; range, 1.3–1.7 mm) away from the seed point were significantly greater than shuffle (*p* < 0.05) in all animals. Error bars show interquartile range.

To assess the statistical significance of long-range correlations ∼1.5 mm from the seed point (Fig. 1*E–G*), we compared correlations with a surrogate control as described previously (Smith et al., 2018). Briefly, we first identified the local maxima (minimum separation between maxima: 800 µm) in the correlation pattern for each seed point. We then compared the median correlation strength for maxima located 1.3–1.7 mm away against a distribution obtained from 100 surrogate correlation patterns.

These surrogate correlation patterns were obtained by eliminating most of the spatial relationship between the patterns. To this end, all activity patterns were randomly rotated (rotation angle drawn from a uniform distribution between 0 and 360° with a step size of 10°) and reflected (with probability 0.5, independently at the *x*- and *y*-axes at the center of the ROI), resulting in an event-matched control ensemble with similar statistical properties but little systematic interrelation between patterns. Surrogate correlation patterns were then computed from these ensembles as described above.

Finally, for each individual animal, the *p* value was taken as the fraction of median correlation-strength values from surrogate data greater than or equal to the median correlation strength for real correlation patterns.

#### Wavelength

To estimate the wavelength of individual calcium events, we calculated the spatial autocorrelation of the Gaussian high-pass filtered events (Powell et al., 2024a). We then took the radial average of the autocorrelation to get a one-dimensional autocorrelation function. The wavelength of the event was calculated as twice the distance to the first minimum from the origin.

**Active area** $\left(A_{\mathrm{ACTIVE}}\right)$ is the number of pixels that were active at any point during the event, multiplied by the area of each pixel ( $A_{\mathrm{PIX}}$, 0.0022 mm^2^). An array of deconvolved activity $y_{\mathrm{pt}}$ contains *N* pixels and *T* frames per event. The ratio active (RA) is the ratio of active area over total area of the ROI $\left(A_{\mathrm{TOTAL}}\right)$:

$$p_{\mathrm{SUM}}=\;\overset{N}{\underset{\;p=1}{\sum }}\;\left(\left(\overset{T}{\underset{t=1}{\sum }}\;y_{\mathrm{pt}}\right)>0\right),$$

$$A_{\mathrm{ACTIVE}}=p_{\mathrm{SUM}}\;*\;A_{\mathrm{PIX}},$$

$$\mathrm{RA}=\;\frac{A_{\mathrm{ACTIVE}}}{A_{\mathrm{TOTAL}}}.\;$$

**Propagation area** (PA) is the area of additional activity after the onset frame activity $\left(A_{\mathrm{ON}}\right)$. In order to mitigate the effects of imaging noise and identify events in which activity showed propagation, we excluded activity immediately surrounding $A_{\mathrm{ON}}$ in a radius of ∼420 µm in the calculation of new active area after the onset frame. We achieved this using a morphological dilation operation, executed by *binary_dilation* from the Python package *scipy*. A square connectivity matrix (3 × 3 matrix where all elements equal 1) was iteratively dilated nine times for a total exclusion radius of ∼420 μm. Thus, PA is equal to $A_{\mathrm{ACTIVE}}$ minus the dilated $A_{\mathrm{ON}}$. Given this exclusion radius, we then set a threshold of PA = 0 for “static” events and classified events with PA > 0 (events with activity extending at least 420 µm beyond $A_{\mathrm{ON}}$) as “dynamic.”

#### Linear wavefront fitting

A linearly traveling wavefront was estimated for each dynamic event (PA > 0) with variables velocity (v), direction (θ), and time shift $\left(t_{\mathrm{SHIFT}}\right)$, similar to Smith et al. (2015a). The $t_{\mathrm{SHIFT}}$ variable is a bias term that shifts where the wave begins. Given a linearly traveling wave with variables $\left\{v,\theta ,\;t_{\mathrm{SHIFT}}\right\}$, the estimated onset times were computed $\left({\hat{t}}_{\mathrm{ON}}\right)$ for each pixel location, where $x_{p}$ is the (x,y) locations of pixel *p.* Only the onset time was modeled. The optimal model parameters ( $\left\{\hat{v},\hat{\theta },{\hat{t}}_{\mathrm{SHIFT}}\right\}$) were found by minimizing the mean squared error (MSE) between ${\hat{t}}_{\mathrm{ON}}$ and the observed onset times $\left(t_{\mathrm{ON}}\right)$, using an initial grid search and then fine-tuning the values using an optimization algorithm:

$${\hat{t}}_{\mathrm{ON},n}=\frac{x_{p}\cdot [\cos\;\theta ,\sin\;\theta ]}{v}+t_{\mathrm{SHIFT}},$$

$$\mathrm{MSE}\;=\;\frac{1}{N}\left(\overset{N}{\underset{n=1}{\sum }}\;{\left(t_{\mathrm{ON},\;n}-{\hat{t}}_{\mathrm{ON},n}\right)}^{2}\right),$$

$$\{\hat{v},\hat{\theta },{\hat{t}}_{\mathrm{SHIFT}}\}\;=\min(\mathrm{MSE},\;\{v,\theta ,t_{\mathrm{SHIFT}}\}).$$

To determine if an event had a linear fit significantly better than chance, we permuted the order of frames for each event and fit the permutations, using the 95th percentile of the permuted data as a threshold for a significant linear fit (Siegel et al., 2012). To ensure enough possible permutations for statistical comparison, we restricted our analysis to events with five or more frames.

To determine the maximum velocity detectable with our imaging parameters, we considered our imaging framerate of 50 Hz and the size of each animal's imaging window. Given a 3-mm-diameter imaging window, activity could be observed to shift over the course of one frame (20 ms) with a maximum velocity of ∼150 mm/s. Thus, any events with fit velocities greater than this maximum value (4% of all events) were excluded from the calculation of the fraction of linearly propagating events.

The estimated propagation directions in our data appeared to have a symmetric bimodal distribution where the activity traveled across the imaging field of view (FOV) roughly along an axis in either direction. To test this, we converted each estimated propagation angle $\left(\theta_{\mathrm{dir}}\right)$ into axial space:

$$\theta_{\mathrm{axial}}=\mathrm{mod}(\theta_{\mathrm{dir}},180).$$

Then we applied the Rayleigh test for significant axial bias. If an animal had a significant axis of propagation, we further tested if there was a symmetric but uneven direction preference by comparing the number of linear events propagating along either direction (using ±90° bins around the mean axis) and performing a binomial significance test.

#### Event repetitions and clustering

To identify repeated spatiotemporal patterns in events (Fig. 4), we first identified a subset of events with the same number of data points (five frames), thereby allowing us to make direct comparisons of the spatiotemporal structure across events. Each event frame was Gaussian bandpass filtered (SD: *s*_low_ = 23 µm, *s*_high_ = 194 µm), in order to emphasize the modular patterns and to mitigate noise. To ensure that our results did not depend on specific filter setting, we did a sweep across various settings (no filters to *s*_low_ = 94 µm and *s*_high_ = 560 µm). The results from Figure 4 were similar across these settings, except for one animal (F337) which had lower SNR and weaker GCaMP expression than other cases. Given the dependence of results from F337 on the choice of filter settings, this animal was excluded from further analysis.

Data was denoised through PCA decomposition (retaining first 20 components), then all frames within an event were concatenated, and we computed the event–event correlation matrix using Pearson’s correlation coefficient, thus capturing both the spatial and temporal components of activity. We determined whether the spatiotemporal structure of an event was repeated with a permutation test based on a random model. Given that events typically showed a general rise and fall in activity across the event, we controlled for this in the model by permuting within a time step, i.e., all the first frames were permuted across events, all second frames, etc. Thus, we took 1,000 within-time step permutations, computed 1,000 correlation matrices, and the 99th percentile of all permuted correlation values was used as a threshold for a significant correlation (Fig. 4*D*). We applied this threshold to determine the number of repetitions per event, with all events showing correlations over this threshold considered as repetitions of a given event. The event frequency was calculated as the number of repetitions divided by the number of total events (Fig. 4*E*).

With these repeated events, we utilized a greedy algorithm to find clusters of patterns. That is, the event with the highest number of repeats defined the first cluster as this set of repeated events. These were excluded on the next iteration and then the second cluster was found, etc. The stop condition for the algorithm was when the clusters no longer had at least 10 events, and thus clusters were included in the following analyses if they included at least 10 events. The fractional range $\left(F_{r}\;\right)$ of each cluster (Fig. 4*I*) was then computed using the initiation times of events (***t***) in that cluster, divided by the duration of the imaging session, $t_{\mathrm{TOTAL}}$:

$$F_{r}=\frac{\max(t)-\min(t)}{t_{\mathrm{TOTAL}}}.$$

The fraction of represented bins $\left(F_{b}\right)$ (Fig. 4*I*) for each cluster was computed as follows:

$$F_{b}=\frac{1}{N}\left(\overset{N}{\underset{n=0}{\sum }}\;b_{i}\right),\;\;b_{i}=\{\begin{array}{c} 0\;\mathrm{if}\;\mathrm{no}\;\mathrm{event}\\ \;1\;\mathrm{if}\;\mathrm{event}\; \end{array},$$

where *N* is the total number of bins (*b*) and each bin is a 10 min segment of the imaging session.

To ensure that our analysis was not dependent on this specific clustering algorithm, we compared the clusters of 100 ms events identified with the greedy algorithm to those found through either hierarchical or *k*-means clustering, using the same number of clusters across algorithms. We first examined how well each clustering algorithm performed. We used the silhouette score, which is a summary of the within-cluster distance versus the between-cluster distance, implemented using the Python library scikit-learn:

$$\mathrm{silhouette}\;\mathrm{score}=\frac{1}{N}\sum \frac{\left(b_{i}-a_{i}\right)}{\max(a_{i},b_{i})},$$

where $a_{i}$ is the mean nearest-cluster distance for a sample and $b_{i}$ is the next nearest-cluster distance for a sample. We compared the silhouette score as a percentage of the performance of the greedy algorithm:

$$\mathrm{silhouette}\;\mathrm{difference}=\frac{\mathrm{silhouett}e_{\mathrm{greedy}}-\mathrm{silhouett}e_{\mathrm{method}}}{\mathrm{silhouett}e_{\mathrm{greedy}}}*100\text{\%}.$$

We also computed the fractional range $\left(F_{r}\;\right)$ and fraction of represented bins $\left(F_{b}\right)$ for both hierarchical and *k*-means clustering (as above) and compared these to the metrics for the greedy algorithm.

In order to confirm that our clustering approach was sensitive to the temporal structure within events (and not only to spatial features), we pseudorandomly permuted the frame order in each event, thereby maintaining spatial structure while removing temporal information. These permutations were performed such that all permuted frames were placed in a different position within the event. We then took the correlation to each cluster and assigned the permuted event either to the cluster with the highest correlation or to a null cluster if it did not pass the threshold for significant correlation as described in above. We then report the percentage of permuted events assigned to their original cluster.

#### Template frame selection and group membership

To identify conserved spatial activity motifs (Fig. 5), we aggregated the frames of all events that were 200 ms or shorter. In order to target modular activity and remove noise, each frame was fit to a two-dimensional multi-Gaussian function, giving us a set of “template patterns.” We computed a threshold for each template pattern by taking correlation values between the template and translated versions of the template, shifted in *x–y* space up to one standard deviation away from the center of the Gaussian fit and used the lowest correlation value of these translations as the threshold.

We then computed the correlation values between each frame of activity and all template patterns. Using a greedy algorithm (similar to above, Event repetitions and clustering), we identified the template pattern with the most correlated frames above threshold, and these frames defined the first group. We removed those events and repeated the procedure to identify the second group, etc. The greedy method is restricted to only using one frame per event, thus, if two frames were matched in a single event, we took the highest correlated frame to the template to determine group identity. To compare across animals, we used the top 7 groups for all animals. This cutoff was chosen to ensure that every group across animals had at least 15 repeats per group and such that every animal used the same number of groups for cross-animal comparisons.

#### Classification accuracy for template groups

We independently decomposed each time point into its first eight principal components. Groups were matched to have the same number of members through subsampling, and we subsampled 100 times. The classification was performed using a support vector machine (SVM) with a radial basis function kernel. To find the reported classification accuracy from −2,000 to 2,000 ms, we took each matched and subsampled set and computed the mean of its fivefold cross-validation test accuracy. This yielded a distribution of classification accuracies for all 100 subsamples.

We used a shuffled dataset to control for any unknown biases in classification accuracy. In order to do this, we took each of the 100 subsamples and permuted group labels for each one 100 times. Each subsample was compared against the 100 permutations of that subsample and was considered significant if classifier performance was greater than the 95th percentile of the permuted data. Finally, a time point was considered classified significantly above chance if 95% of subsamples were classified significantly above chance.

#### Code and software

Preprocessing code is custom and written using Python 3.8. Most of the analysis code uses Python 3.11. We used the following packages extensively for analysis: numpy, pandas, scikit image, scikit learn, scipy, and tifffile. We used pycircstat2 for circular statistics (Fig. 3). We used matplotlib, seaborn, and distinctipy for graphing and accessible colormaps. ImageJ was used to create extended data movies. All code is available upon request.

## Results

### Early cortical activity exhibits fast temporal dynamics

In order to examine the spatiotemporal structure of millimeter-scale modular spontaneous activity in early development, we injected the neonatal ferret cortex with AAV expressing GCaMP8m (Zhang et al., 2021) between ages P10 and P14. We then performed wide-field imaging of spontaneous activity between ages P23 and P28, ∼4–9 d before normal eye opening, capturing several hours of activity over approximately a 6 mm^2^ FOV (Table 1). Experiments were performed under light isoflurane anesthesia, which in prior work preserves the millimeter-scale spatial structure of spontaneous activity seen in the awake cortex (Smith et al., 2018). For our purposes, this imaging regime also has the advantage of producing spontaneous events that tend to be separated by periods of relatively low activity, allowing us to more easily separate events in order to focus on the fast temporal dynamics within events.

**Table 1.** Experimental information

Ferret ID	Injection age	Imaging age	Expression time (days)	FOV area (mm^2^)	Imaging time (minutes)
F261	14	24	10	5.5	130
F317	12	23	11	5	150
F335	11	23	12	6	165
F336	11	25	14	6.5	165
F337	11	28	17	8.9	135
F339	11	25	14	5.8	145

In order to capture the temporal dynamics of spontaneous activity, we imaged GCaMP8m with its relatively fast decay kinetics (Zhang et al., 2021) at 50 Hz and performed a prior frame subtraction deconvolution method (Stern et al., 2020; Fig. 1*A–C*; see Materials and Methods), allowing us to measure the temporal structure of activity with a resolution of 20 ms. We observed frequent spontaneous events (Fig. 1*A*) that consisted of spatially segregated regions (modules) of activation distributed across the cortical surface (Fig. 1*C*,*D*; Extended Data Movie 1-1). The spatial structure of these patterns was largely similar to that reported previously during early development in V1 with slower calcium sensors (Smith et al., 2018). Notably, many single frames within these events exhibited spatially distributed patterns of activity consisting of multiple active modules (Fig. 1*C*,*D*), suggesting that millimeter-scale modular activity patterns are present within spontaneous activity at a time resolution of 20 ms. However, our fast imaging also revealed previously unappreciated temporal dynamics within spontaneous events, with the spatial patterns of activated modules often shifting over several tens of milliseconds (Fig. 1*D*, top two rows). In other cases, large patterns of active modules appeared nearly simultaneously, with the same spatial pattern maintained over the duration of the event (Fig. 1*D*, bottom).

**Movie 1-1.** Four events are played consecutively after each other. Each event is preceded by a blank title frame, followed by appx 100 ms of activity where event onset time is at 0 ms. Data shown is deconvolved with a mild gaussian lowpass filter. Similar sequential, modular behavior was seen across animals.

We next sought to determine whether the distributed modular activity patterns observed on single frames in our data set are consistent with the millimeter-scale correlated networks reported previously on slower timescales (Smith et al., 2018). To test this, we first computed the spatial wavelength of active modules within an event, finding wavelengths (median, 0.69 mm; range, 0.58–0.77 mm; *n* = 6 animals) similar to those reported previously both for spontaneous activity (0.76–0.86 mm; Powell et al., 2024b) and for orientation preference maps (0.72–0.9 mm; Chapman et al., 1996; Rao et al., 1997; Kaschube et al., 2010). To directly assess correlated networks within our high temporal resolution data, we computed pixel-wise spatial correlations across the single maximally active frame from each spontaneous event, revealing millimeter-scale patterns of positively correlated modules (Fig. 1*E*, left). Notably, the spatial pattern of correlations in single-frame data was highly similar to that obtained after summing all frames within each event (Fig. 1*E*, right), which mimicked the slower frame rates of prior studies. In order to assess the strength of these long-range single-frame correlations, we compared them with shuffled surrogate controls (see Materials and Methods), finding statistically significant correlations out to 1.5 mm (Fig. 1*F*,*G*). Thus, our results show that at fast timescales (20 ms), spontaneous activity in the developing cortex exhibits modular and distributed millimeter-scale organization.

### Spontaneous activity frequently exhibits dynamic propagation across the cortical surface

**Figure 2.**
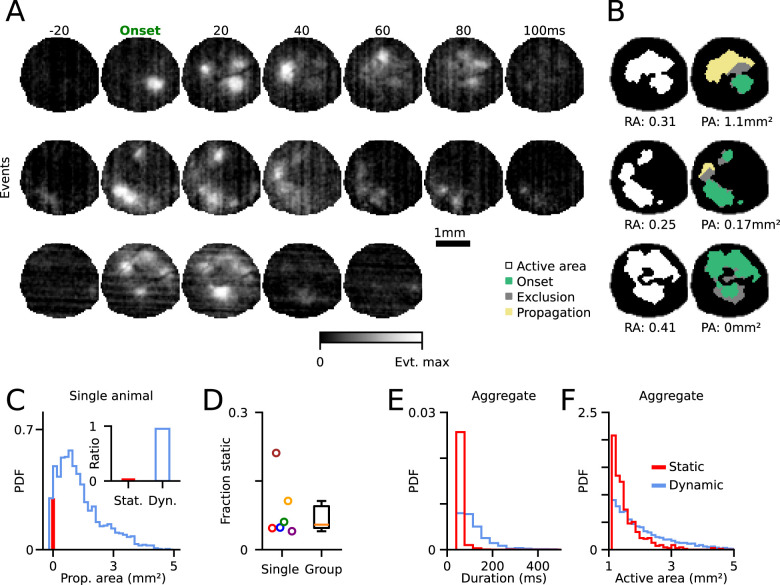
Spontaneous events exhibit propagating activity patterns across the cortex. ***A***, Three example events with varying amounts of propagation. The top two events are dynamic, and the bottom event is static. ***B***, Left, Ratio of all active pixels during entire event (RA). Right, Area of propagating activity (PA, yellow) extending beyond onset activation (green; see Materials and Methods). ***C***, Propagation area across all events. Red bar indicates static events (PA = 0). Inset shows percentage of static and dynamic events (PA > 0) for one animal. ***D***, Summary of static event percentages across animals (mean, 6.9%; range, 4–21%; *n* = 6; example animal is blue). ***E***, Static events exhibit shorter durations than dynamic events and ***F***, static events exhibit smaller active area than dynamic events.

To examine the spatiotemporal properties of the events, we first quantified the degree of spatial propagation within each event. We defined the propagation area (PA) to be the area of active pixels in our imaging FOV extending at least 420 µm beyond the activity from the first (onset) frame of the event (see Materials and Methods; Fig. 2*A*,*B*). This exclusion radius was chosen to mitigate the impact of small fluctuations potentially reflecting imaging noise and allow us to isolate spontaneous events with activity moving across the cortex. A large majority of events showed extensive propagation, with new activity beyond onset patterns frequently extending over 1 mm^2^ (median, 0.80 mm^2^; 25–75th, 0.335–1.399 mm^2^ for 8,093 events from 6 animals; Fig. 2*C*).

To estimate the fraction of events that showed within-event spatial propagation, we categorized all events with PA > 0 (events with activity extending beyond the 420 µm radius) as “dynamic” and propagating and all events with PA = 0 as “static” and nonpropagating. In static events, all activity remained localized to regions present in the initial onset of activity. Across all animals, we found that the majority of events were dynamic and only a small subset was static (mean, 6.9% static; range, 4–21%; *n* = 6 animals; Fig. 2*D*). Note that the requirement that activity must extend at least 420 µm beyond the onset area to be classified as “propagating” means that this is likely a conservative estimate of the fraction of propagating events. This indicates that dynamic events are the prevalent mode of cortical activity at this stage of development. The high frequency of dynamic propagation we observe in our data also argues that our imaging resolution is sufficient to capture the underlying temporal structure of spontaneous activity, as propagation faster than our imaging speed would appear as “static,” which we rarely observe. We next sought to quantify the temporal duration and total active area of these spontaneous events. Overall, static events were significantly shorter in duration than dynamic events (dynamic: median, 100 ms; median range, 60–120 ms; static: median, 40 ms; median range, 40–40 ms; *n* = 6 animals; Mann–Whitney *U* test; *p* = 9.8 × 10^−4^; Fig. 2*E*). Static events also engaged less cortical area (dynamic: median, 1.8 mm^2^; median range, 1.6–2.0 mm^2^; static: median, 1.4 mm^2^; IQR = 1.3–1.4 mm^2^; Mann–Whitney *U* test; *p* = 2.1 × 10^−3^; *n* = 6 animals; Fig. 2*F*). These results indicate that spontaneous events often initiate within a subset of the cortical network and then progress into additional spatial locations. Furthermore, events rarely emerge simultaneously across a large spatial area, but rather expand in footprint dynamically across time.

### Linear traveling waves describe a minority of propagating spontaneous events

**Figure 3.**
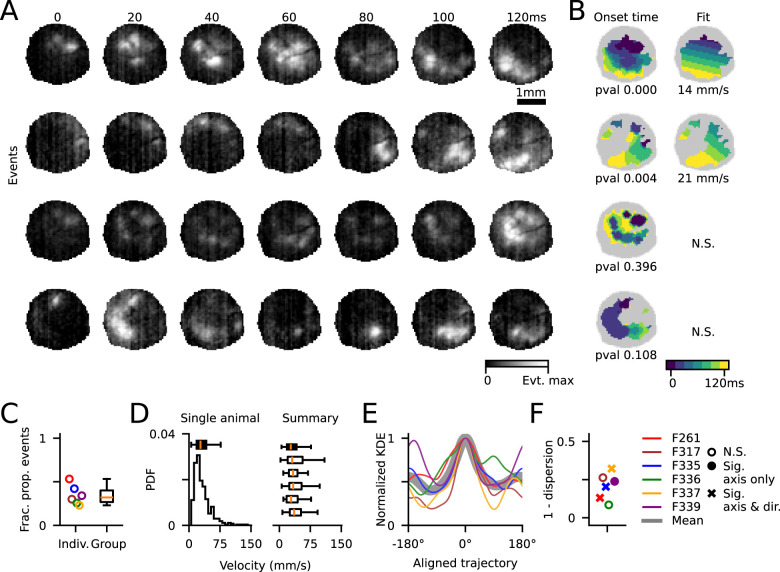
A subset of dynamic events is well described by a linear traveling wave. ***A***, Four example dynamic events. ***B***, Left, Onset time for all active pixels in an event and (right) the linear fit. Bottom two events were not well fit by a linear wave. ***C***, Fraction of events exhibiting significantly linear propagation (median, 32%; range, 22–53%; *n* = 6 animals). ***D***, Wave propagation velocity estimates for linear waves. Left, Example animal and right, all animals. ***E***, Summary of trajectories across all animals, aligned to most common propagation direction and normalized within animal. Black shaded line is the mean across animals. ***F***, 1, Angular dispersion of wave propagation axis for linear waves in each animal. 0 indicates uniform distribution of propagation, 1 indicates same propagation angle for all waves. Distributions were tested for significant axis of propagation (180° space) and if they had a preferred trajectory along the axis. Statistically significant axis of propagation with no preferred direction is shown with a filled circle (*p* < 0.05, Rayleigh test), and axes with preferred trajectory are marked with an “x” (*p* < 0.05, binomial test).

We next examined if this propagating activity moved across the cortical surface in a traveling wave, as has been observed in prior studies (Grinvald et al., 1994; Benucci et al., 2007; Nauhaus et al., 2009, 2012; Sato et al., 2012; Muller et al., 2014). For all dynamic events (PA > 0), we first identified active pixels on each frame of an event, thereby generating a mask of activity. We then fit the activation time of pixels within this mask with a linear traveling wave moving across the imaging FOV. To assess the quality of these linear fits, we compared them with fits of frame-permuted events, thereby maintaining spatial characteristics but destroying temporal relationships (Siegel et al., 2012). We found that across animals, only a minority of dynamic events were well described by a propagating linear wave (median, 32%; range, 22–53%; *n* = 6 animals; Fig. 3*A–C*; Extended Data Movie 3-1).

**Movie 3-1.** Event showing linear wave-like propagating activity (see Fig. 3). Data shown is deconvolved with a mild gaussian lowpass filter.

Of events that were significantly fit by a linear wave, the propagation velocity was consistent across animals, with a median velocity of 32 mm/s (5–95th, 12–114 mm/s, 1,432 events across 6 animals; Fig. 3*D*). Linearly propagating events traveled across a range of directions within each animal. In most animals, however, there was a weak but significant bias in preferred direction (Fig. 3*E*). Four of six imaged animals exhibited a significant axial bias (180°) in linear wave propagation direction (*p* < 0.05, Rayleigh test; Table 2), three of which also exhibited a significant directional bias (post hoc binomial test, *p* < 0.05; Fig. 3*F*). Thus, despite the high fraction of spontaneous events that exhibit fast propagation on the scale of hundreds of milliseconds, relatively few of these events exhibit simple linear dynamics. Rather, the majority of early spontaneous events show more complex and nonlinear spatiotemporal trajectories during early development.

**Table 2.** Linear wave statistics

Ferret	Total events	Significant linear wave	Fraction significant linear wave	Axial (180) Rayleigh test *p* value	Binomial test *p* value
261	867	462	0.53287	0.0001167	3.66781 × 10^−05^
317	100	30	0.3	0.1615	-
335	934	396	0.42398	8.043 × 10^−10^	0.000370051
336	627	161	0.25678	0.4107	-
337	441	102	0.23129	1.089 × 10^−07^	0.000996403
339	824	281	0.34102	6.248 × 10^−10^	0.360233
All	3,793	1,432	0.37754	-	-

### Spontaneous activity shows stereotyped spatiotemporal patterns that are conserved across hours

**Figure 4.**
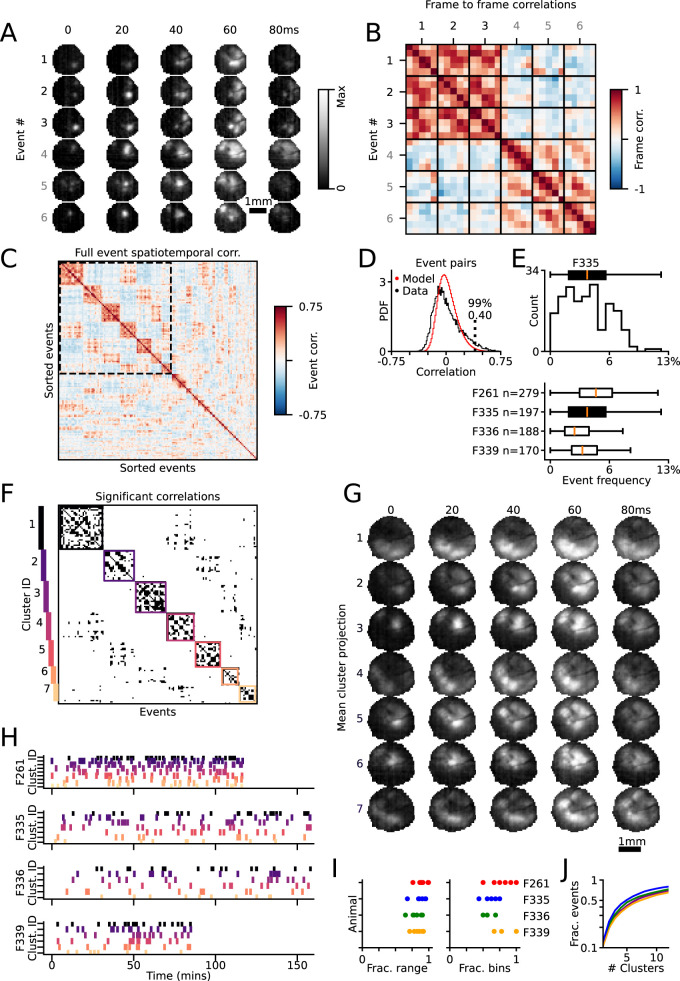
Spatiotemporal patterns within events are persistent across hours. ***A***, Two example event clusters show two distinct spatiotemporal patterns. Both event clusters initiate with a single module and progress into multiple activated modules. ***B***, Frame-to-frame correlations of events in ***A***. There is high correlation within clusters and negative correlation between clusters. Additionally, there is higher correlation along diagonals, reflective of shared dynamic activity across time. ***C***, Clustered correlation matrix of all 100 ms events in example animal. ***D***, Distribution of event–event correlations for actual (black) and random model (red; see Materials and Methods). Threshold for significant correlations (99th percentile of model) indicated with dashed line. ***E***, Top, Distribution of event repeat frequency for all 100 ms events, for example, animal shown in ***A–D*** and (bottom) for all animals. ***F***, Thresholded and zoomed-in version of correlation matrix in ***C***. Color-coded boxes drawn around identified clusters. ***G***, The spatiotemporal clusters that passed the event number threshold for animal F335. Each row is the mean projection of events in a cluster, showing the progression across five frames. ***H***, Initiation times of events found in each cluster, where each row is a separate animal. ***I***, Left, Fraction of recording time (fractional range) spanned by instance of repeated event motifs. Right, Fraction of 10 min bins (fractional bins) with at least one instance of a repeated event. Each dot indicates data for one repeated motif. ***J***, Fraction of events included as a function of increasing cluster number.

In order to better understand the complex spatiotemporal dynamics of spontaneous activity in the early developing cortex, we next examined if similar spatial and temporal patterns of activation occurred across multiple events. To achieve this, we first identified dynamic events (PA > 0; see Materials and Methods) with event durations of 100 ms, allowing for the direct comparison of aligned frames across events. We then computed event–event correlations by combining all active frames within each event, allowing us to capture the overall similarity in activation patterns while incorporating both spatial and temporal components. To illustrate this approach, we selected two dynamic sets of events that showed high event-level correlations within sets and low correlations between sets (Fig. 4*A*; Extended Data Movie 4-1). We then computed the spatial frame-to-frame correlations for all frames across these events (Fig. 4*B*). This revealed high correlations between aligned frames across correlated events, as illustrated by the structure of large, positive correlations in the off-diagonal elements of the frame–frame correlation matrices across events (Fig. 4*B*, compare event 4 to events 5 and 6). Such correlation structure indicates that the spatial pattern of activity followed a similar progression across time in each event.

**Movie 4-1.** Movie showing two sets of spatiotemporal patterns. Each row corresponds to a cluster as defined in Fig. 4. Each event is labeled with their initiation time in minutes from the start of the session, the same as in Fig. 4H.

To extend this observation across multiple events, we used a clustering approach to identify similar spatiotemporal activity patterns across events. Specifically, we computed the event–event correlation matrix across all events with duration of five frames (100 ms) and identified clusters of highly correlated events using a greedy algorithm (see Materials and Methods), which revealed subsets of spatiotemporally distinct event patterns (Fig. 4*C*). We then thresholded these correlations using the 99th percentile of a control shuffle distribution to identify significant correlations (Fig. 4*D*). To estimate how often a given spatiotemporal event pattern showed significant similarity to the patterns of activity on other events, we then applied this threshold for each event to determine the number of repetitions per event, considering all other events with correlations greater than this threshold as “repetitions.” The event frequency was then calculated as the number of repetitions divided by the number of total events. We found that events had a median repetition frequency of 3.7% (25–75th, 2.4–5.7%; *n* = 834 across 4 animals; Fig. 4*E*), and 98.0% of events were repeated at least one time (*n* = 834 events across 4 animals). Furthermore, a large fraction of 100 ms events were contained within the six largest identified clusters (Fig. 4*J*; median, 51%; range, 44–62%; *n* = 4 animals), indicating that many events occurred as repeated instances of similar spatiotemporal patterns, and consistent with the moderately low dimensionality observed previously in the spatial patterns of spontaneous events (Smith et al., 2018). To verify that our clustering was sensitive to temporal order, we performed a frame-shuffled control and found that in each cluster, only a small fraction of frame-shuffled events maintained their cluster membership (median, 4.1%; IQR = 2.4–5.3%; *n* = 28 clusters from 4 animals; see Materials and Methods).

We next examined the temporal distribution of repeated instances of similar spatiotemporal event patterns across the hours of imaging in our dataset. Using the most frequently observed clusters of similarly structured events (min 10 events per cluster; Fig. 4*F*,*G*), we observed that instances of each cluster occurred throughout the majority of the imaging session (Fig. 4*H*, Extended Data Movie 4-1). Across animals, significantly similar events were observed spanning hours of imaging, both covering a large fraction of time within an imaging session (percentage imaging time spanned by event instances, fractional range $\left(F_{r}\;\right)$: median, 89%; 25–75th, 81–91%; *n* = 28 clusters across 4 animals; Fig. 4*I*, left) and occurring within the majority of 10 min segments of binned spontaneous activity (percentage segments with identified event, fraction of represented bins $\left(F_{b}\right)$: median, 67%; 25–75th, 58–83%; *n* = 28 clusters across 4 animals; Fig. 4*I*, right). Notably, these findings did not depend on the algorithm used to cluster events, as both hierarchical and *k*-means clustering yielded similar clustering performance (silhouette difference, as a percentage of the greedy clustering silhouette score: hierarchical, median, 4%; range, −2–14%; *k*-means, median, 0.5%; range, −9–4%; *n* = 4 animals) and similar distributions of clustered event instances over time ( $F_{r}\;$: hierarchical, median, 87%; 25–75th, 82–91%; *k*-means, median, 88%, 25–75th, 83–94%. $F_{b}$: hierarchical, median, 74%; 25–75th, 60–83%; *k*-means, median, 69%; 25–75th, 60–85%). Collectively, these analyses demonstrate that spontaneous activity in the developing cortex contains distinct motifs of spatiotemporal activity patterns, which occur repeatedly across hours of cortical activity.

### Spontaneous activity motifs are predictive of future activity patterns over hundreds of milliseconds

One of the most prominent features of early spontaneous activity is the presence of long-range correlations, indicating that certain subsets of spatial modules tend to be repeatedly engaged across events (Smith et al., 2018). This is supported on a finer timescale by our finding of repeated spatiotemporal motifs in spontaneous activity (Fig. 4) and by the presence of long-range correlations at single-frame timescales (Fig. 1*E–G*). This raises the possibility that activity within an event propagates along a limited subset of distinct spatiotemporal trajectories, reflecting sets of modules within the network. Alternatively, the trajectories of spontaneous events could intermingle, and any given pattern of activity within an event could lead to a broad range of potential subsequent patterns. Therefore, in addition to considering the full spatiotemporal similarity of activity over an event (Fig. 4), we sought to investigate the degree to which the instantaneous activity at a single time point could provide information about the structure of both preceding and subsequent activity.

**Figure 5.**
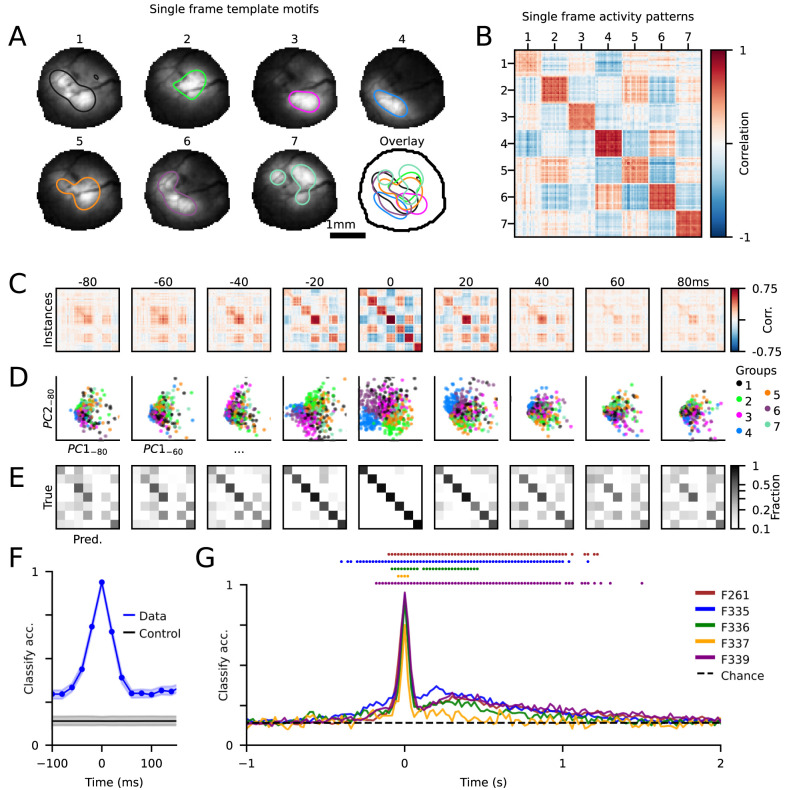
Spontaneous activity motifs are predictive of future activity patterns over hundreds of milliseconds. ***A***, Template frame motifs, showing the average single-frame group activity, identified by finding highly repeated instances in single frames of activity. Contours are drawn to illustrate each template's active area. ***B***, Single-frame correlation matrix, sorted by group. ***C***, Single-frame correlation matrices for time points −80 to +80 ms, from template groups sorted as in ***B***. ***D***, Group separability shown for all time points, plotted as first two principal components of each time point. ***E***, Group classifier performance from −80 to +80 ms. ***F***, Classification accuracy across time for example animal shown in ***A–E*** and control scores’ mean performance of shuffled groups (CI = 5–95%). ***G***, Classification accuracy for all animals. Dots indicate time points for each animal significantly above shuffled control accuracy (*p* < 0.05).

We reasoned that by identifying highly repeated single-frame activity patterns, we would be able to determine if spontaneous events follow a consistent trajectory through activity states or if conserved single-frame activity patterns could be engaged from many different trajectories. To this end, we took all events with duration ≤200 ms and first identified for each animal template motifs based on the seven most common single-frame activity patterns (Fig. 5*A*; see Materials and Methods). We then grouped these events based on their single-frame similarity to these template motifs, with the group label determined by the best matching template (see Materials and Methods). Comparing frame-to-frame correlations both within and across groups revealed a clear separation in activity patterns, as expected (Fig. 5*B*). Within these grouped events, we found many 100 ms long events, and in fact many of the 100 ms events that exhibited significant spatiotemporal correlations on a whole event level (Fig. 4*D*) also contained a template motif (median, 50% of these 100 ms events; range, 30–57%; *n* = 4 animals). However, because our analysis in Figure 4 generated clusters of events based on the spatiotemporal correlations over the full duration of 100 ms events, the identified clusters in Figure 4 do not directly correspond to the single-frame groups considered here.[Fig JN-RM-1420-24F6]

**Figure 6. JN-RM-1420-24F6:**
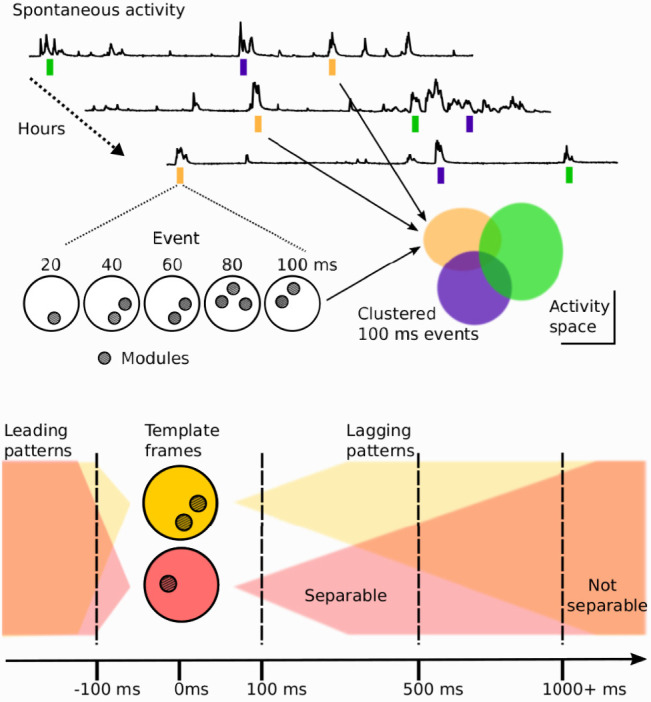
Summary of spontaneous spatiotemporal activity in neonatal ferret visual cortex. Top, Spatiotemporal patterns in spontaneous activity cluster into discriminable activity spaces, demonstrating stereotyped behavior that repeats across hours. This highly organized activity suggests a potential role in the maturation and refinement of future functional representations. Bottom, Single-frame spontaneous activity patterns inform lagging activity patterns up to 1,000 ms and are influenced by leading activity patterns by up to 100 ms. The tendency of cortical activity to converge on a relatively small number of states and retain state information suggests that the system is possibly in an attractor regime.

To understand how these patterns of activity change over time, we examined the frame-to-frame correlations for each event at each time steps up to ±80 ms away from the template frame using the group labels defined at the template frame (*t* = 0). The strong organization in the correlation matrices when sorted by group label appeared to retain some structure even ±80 ms away, as evidenced by the prevalence of positive correlations near the diagonal (Fig. 5*C*). To understand how groups remained separable across time, we projected activity patterns into the principal component space computed at each time step. Single events are plotted by their coordinates in the first two PCs and colored by the group label at *t* = 0 (Fig. 5*D*, Extended Data Movie 5-1). The PCA projections suggest strong organization around *t* = 0, consistent with the sorted correlation tables, with increasing across-group overlap becoming apparent at more distant time points (Fig. 5*D*).

**Movie 5-1.** Movie showing a subset of groups (Fig. 5) and their projection into PCA space. For better visualization, all time points use the same PCA space from time=0ms. The top left panel in the movie shows individual events and the projection in the first three principal components across time. The right panel shows the group average for three groups and a shaded region representing one standard deviation away. The bottom panel shows classification accuracy across time for this example animal (see Fig. 5G).

To quantify this separability between activity trajectories and to determine the degree to which they diverge over time, we trained an SVM classifier to predict the group label (defined at *t* = 0) at each time step up to ±2,000 ms away, using the first eight principal components of events as the input (see Materials and Methods). Confusion matrices, which report the fivefold cross-validation test accuracy, revealed that although classifier performance drops off rapidly with time, it remains above chance levels for at least many tens of milliseconds within an event (Fig. 5*E*), which was confirmed by comparing classifier accuracy to shuffled control data (Fig. 5*F*). Across most animals, we found that statistically significant classifier performance extended well beyond the template frame, including up to 1 s later (Fig. 5*G*). Notably, although the classifier was able to predict group identity for frames preceding the template in all cases, classifier performance exhibited a pronounced asymmetry with respect to time and had significant accuracy extending much further in time following the template. This suggests that after passing through an activity state highly similar to a template—which were identified as the most common single-frame activity patterns—the space of trajectories of spontaneous activity becomes narrower and more conserved, thereby leading to significant classifier performance over long timescales. Collectively, these results indicate that the complex and largely nonlinear patterns of propagating activity within spontaneous events are not random but instead tend to propagate along conserved spatiotemporal trajectories.

## Discussion

We imaged mesoscopic spontaneous activity in the developing ferret visual cortex at high temporal resolution, revealing the dynamic properties of spontaneous network events. We found that nearly all events exhibited fast temporal dynamics, with activity patterns shifting over tens of milliseconds within an event, while showing distributed modular activity on fine timescales. In most cases, this dynamic activity exhibited complex spatiotemporal patterns and was poorly described by a linear wave propagating through spatial modules. Intriguingly, we observed a high degree of stereotypy in individual events, with more than half of events repeating multiple times throughout the recording. Furthermore, spatiotemporal activity patterns exhibited conserved trajectories over long timescales, with network events retaining information about past frames for as long as a second.

Our study builds on previous work that characterized the spatial correlations of the early neonatal cortex, while revealing previously unappreciated temporal dynamics. In the visual cortex (Smith et al., 2018) as well as in other cortical areas early in development (Powell et al., 2024a), spontaneous activity is typified by distributed, modular patterns, with correlated activity across millimeters. Based on the ability of early spontaneous correlations to predict the future visually evoked orientation preference map (Smith et al., 2018), this correlated activity has been proposed to contribute to the formation of functional sensory representations (Smith et al., 2018; Trägenap et al., 2023). Our finding that significant correlations are present between modules over millimeters with fast temporal resolution is consistent with such a role, as the 20 ms temporal resolution of single-frame correlations in our data is well within the temporal range for many forms of synaptic plasticity (Caporale and Dan, 2008). This is further supported by the similarity in spatial correlation structure between single-frame (20 ms) and temporally summed activity (analogous to the slower frame rates of prior studies) in our data (Fig. 1*E*, left vs right), suggesting that fast timescale spatiotemporal correlations in spontaneous activity may contribute to future function. However, future studies employing longitudinal imaging at fast timescales will be required to explicitly address this.

In the visual cortex, local recurrent intracortical connections are thought to give rise to large-scale modular activity patterns through self-organizing mechanisms (Mulholland et al., 2024b). Such a mechanism is supported both by the ability of the cortex to transform uniform optogenetic stimulation into modular activity patterns (Mulholland et al., 2024b) and also by the persistence of millimeter-scale correlated activity following the disruption of feedforward inputs (Chiu and Weliky, 2001; Smith et al., 2018). Furthermore, computational models implementing local excitation/lateral inhibition models (LE/LI) are able to recapitulate the spatial characteristics of millimeter-scale correlation patterns using only local connections (Smith et al., 2018), consistent with the immaturity of long-range anatomical connectivity at this developmental stage (Durack and Katz, 1996) and the presence of tightly coupled excitatory and inhibitory networks at this stage of development (Mulholland et al., 2021). Although such LE/LI networks can readily generate dynamic modular activity in response to transient inputs, future work will be required to determine the degree to which networks implementing this LE/LI framework can also recapitulate the stereotyped fast temporal dynamics we observe in vivo. Combining such computational modeling with recently developed dual color voltage indicators (Kannan et al., 2022) may allow the direct observation of both excitatory and inhibitory activity with millisecond timescales, potentially providing a critical test of LE/LI interactions in the early cortex.

Wave-like propagation of cortical activity has been observed in a number of studies including adult cats and monkeys (Grinvald et al., 1994; Benucci et al., 2007; Nauhaus et al., 2009, 2012; Sato et al., 2012; Muller et al., 2014).These results contrast with our finding that the majority of events were poorly described by a linear wave and exhibited more complex spatiotemporal dynamics, potentially reflecting differences in early development versus the mature cortex. Waves observed in the mature visual cortex have faster wavefronts than our observations (Benucci et al., 2007; Muller et al., 2014), thus likely relying on horizontal connections (Muller et al., 2018) which are yet immature in our ferrets (Durack and Katz, 1996). Differences in recording modalities may also account for these results, as electrophysiological and voltage-sensitive dye measures of activity are sensitive to subthreshold changes in activity, while calcium imaging is more sensitive to suprathreshold events. Wave-like activity has also been observed in local populations of neurons with two-photon imaging in the mouse (Siegel et al., 2012) and the ferret (Smith et al., 2015a), although given the differences in imaging FOV, direct comparison to our mesoscopic data is difficult. Interestingly, more complex nonlinear waves, such as spiral waves, have been reported in cortex (Huang et al., 2010), but with the short duration and modular structure of the events we observe, an analysis of spiral structure in our dataset is likely to be inconclusive.

While the spatiotemporal patterns of spontaneous activity we observed were complex, the trajectories of propagating activity were strongly nonrandom. Approximately half of the 100 ms spontaneous events we observed could be well described by a relatively small number (<6) of stereotyped sets of activity patterns that recurred across hours of spontaneous activity (Fig. 6). Similarly structured event–event correlation matrices have been observed in in vitro networks of cultured neurons that exhibit avalanche criticality (Beggs and Plenz, 2004). A common network-level model may account for this behavior (Morrell et al., 2024), but that remains to be explored in future work. Intriguingly, when we identified the most frequently observed spatial patterns, we found that activation of a particular spatial motif was predictable up to 100 ms prior to activation and the subsequent trajectory retained information about motif identity for nearly 1,000 ms following activation (Fig. 6). The tendency of cortical activity to converge on a relatively small number of states and retain state information suggests that the system is possibly in an attractor regime (Miller, 2016). Attractor dynamics have been used to model mature visual cortex, including area MT (Pattadkal et al., 2024) and V1 (Priebe, 2016), with our results suggesting they may also apply to early development at the modular scale. The use of patterned optogenetic stimulation (Mulholland et al., 2024a) may provide a means to directly test such a role for attractor dynamics in the stereotyped spatiotemporal structure of spontaneous activity.

In summary, our results reveal for the first time the complex spatiotemporal patterns of spontaneous activity in the early developing visual cortex. Even at this early stage of development, highly dynamic activity patterns are structured and nonrandom, with stereotyped spatial patterns that evolve in temporally predictable patterns. Additionally, such fine-timescale stereotyped spatiotemporal patterns may reflect a common feature of developing sensory systems, in which low-dimensional precursors set the stage for later sensory processing. For example, propagation biases in spontaneous stage 2 retinal waves (which occur at ages earlier than those examined in Huberman et al., 2008) appear to prepare the visual system for movement-induced optic flow that occurs following eye opening (Ge et al., 2021; Tiriac et al., 2022). Thus, the structured sequences we observe in spontaneous activity may suggest mechanisms through which early activity patterns might support the development and refinement of mature functional networks.
